# Supplementary material for: ATF7IP-Mediated Stabilization of the Histone Methyltransferase SETDB1 Is Essential for Heterochromatin Formation by the HUSH Complex
Source: Cell Rep. 2016 Oct 11;17(3):653–9. doi: 10.1016/j.celrep.2016.09.050 (PMC5081395; doi:10.1016/j.celrep.2016.09.050)
Supplement: Document S1. Supplemental Experimental Procedures, Figures S1–S4, and Tables S1 and S2 [file mmc1.pdf]

Cell Reports, Volume *17*

Supplemental Information

ATF7IP-Mediated Stabilization of the Histone Methyltransferase SETDB1 Is Essential for Heterochromatin Formation by the HUSH Complex

Richard T. Timms, Iva A. Tchasovnikarova, Robin Antrobus, Gordon Dougan, and Paul J. Lehner

SUPPLEMENTAL FIGURES

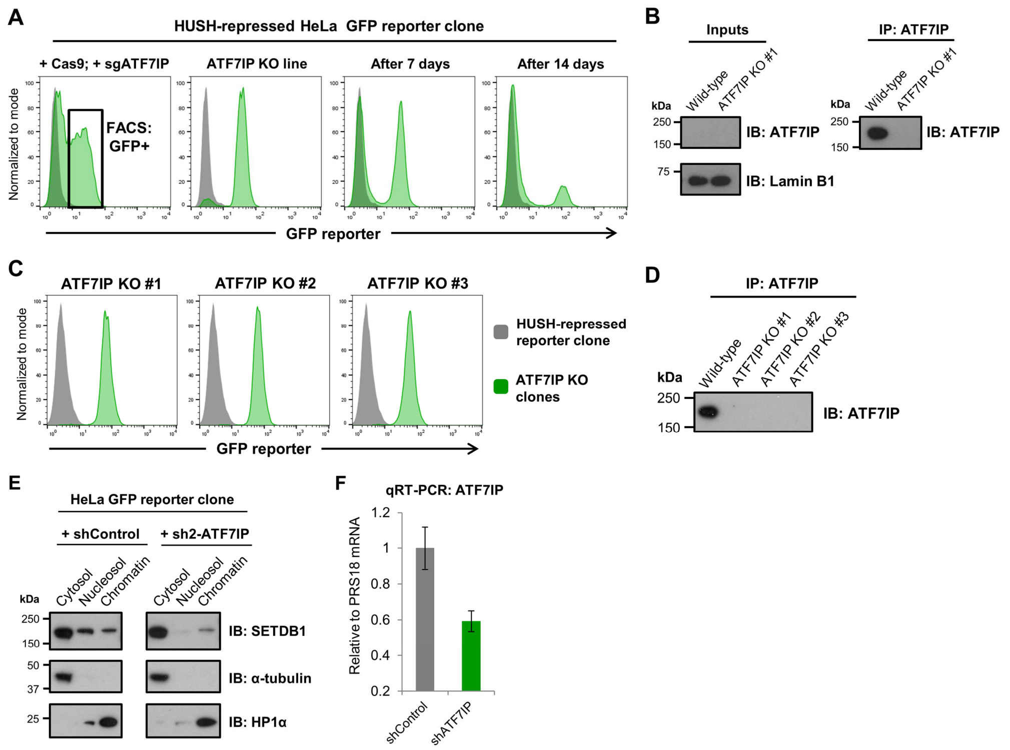

Figure S1. Generation of ATF7IP knockout HeLa clones through CRISPR/Cas9-mediated gene disruption, Related to Figures 1 and 2.

(**A**) A HeLa clone harboring a HUSH-repressed GFP reporter was transfected with plasmids encoding Cas9 and a pool of three sgRNAs targeting ATF7IP. The resulting GFP^{+} cells exhibiting functional loss of ATF7IP were isolated by FACS, yielding a polyclonal population of ATF7IP knockout cells of ~95% purity. The ATF7IP knockout cells in the population were rapidly overgrown by the remaining wild-type cells, however, necessitating the derivation of individual single cell ATF7IP knockout clones.

(**B-D**) Validation of the ATF7IP knockout HeLa clones. Our antibody against ATF7IP (Bethyl Laboratories, A300-169A) was unable to detect ATF7IP protein in wild-type cells in direct immunoblot analysis; however, we were able to detect ATF7IP by immunoblot following a prior immunoprecipitation step (B). We used this immunoprecipitation-immunoblot assay to verify a loss of ATF7IP protein expression in three independent GFP^{+} ATF7IP knockout clones (C,D).

(**E-F**) Destabilization of nuclear SETDB1 following shRNA-mediated depletion of ATF7IP. HeLa cells were transduced with an expression vector encoding either a control shRNA or an shRNA against ATF7IP, and SETDB1 levels were assessed by immunoblot following subcellular fractionation (E). Knockdown efficiency was assessed by quantitative reverse transcription PCR (qRT-PCR) (F); error bars represent the standard deviation of three qPCR technical replicates.

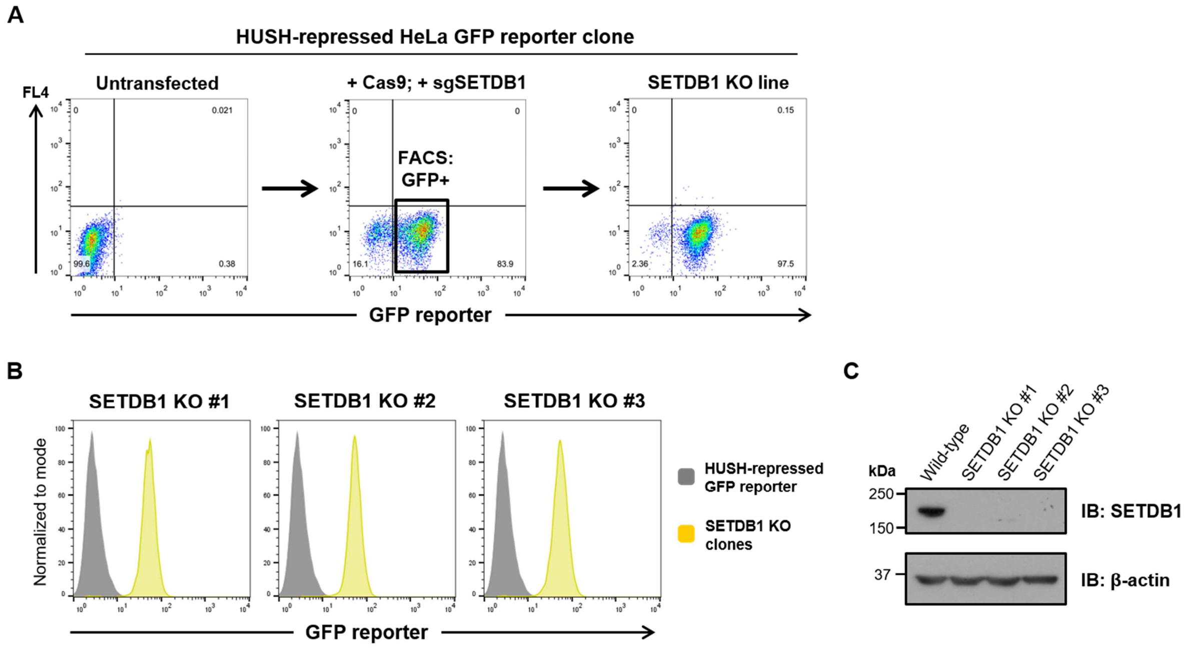

Figure S2. Generation and validation of SETDB1 knockout clones, Related to Figure 2.

(**A,B**) Generation of SETDB1 knockout clones through CRISPR/Cas9-mediated gene disruption. A HeLa clone harboring a HUSH-repressed GFP reporter was transfected with plasmids encoding Cas9 and a pool of four sgRNAs targeting SETDB1. The resulting GFP^{+} cells exhibiting functional loss of SETDB1 were isolated by FACS, yielding a population of SETDB1 knockout cells (A) from which three independent GFP+ single cell clones were derived (B).

(**C**) Immunoblot validation of a lack of SETDB1 protein expression in the three knockout clones.

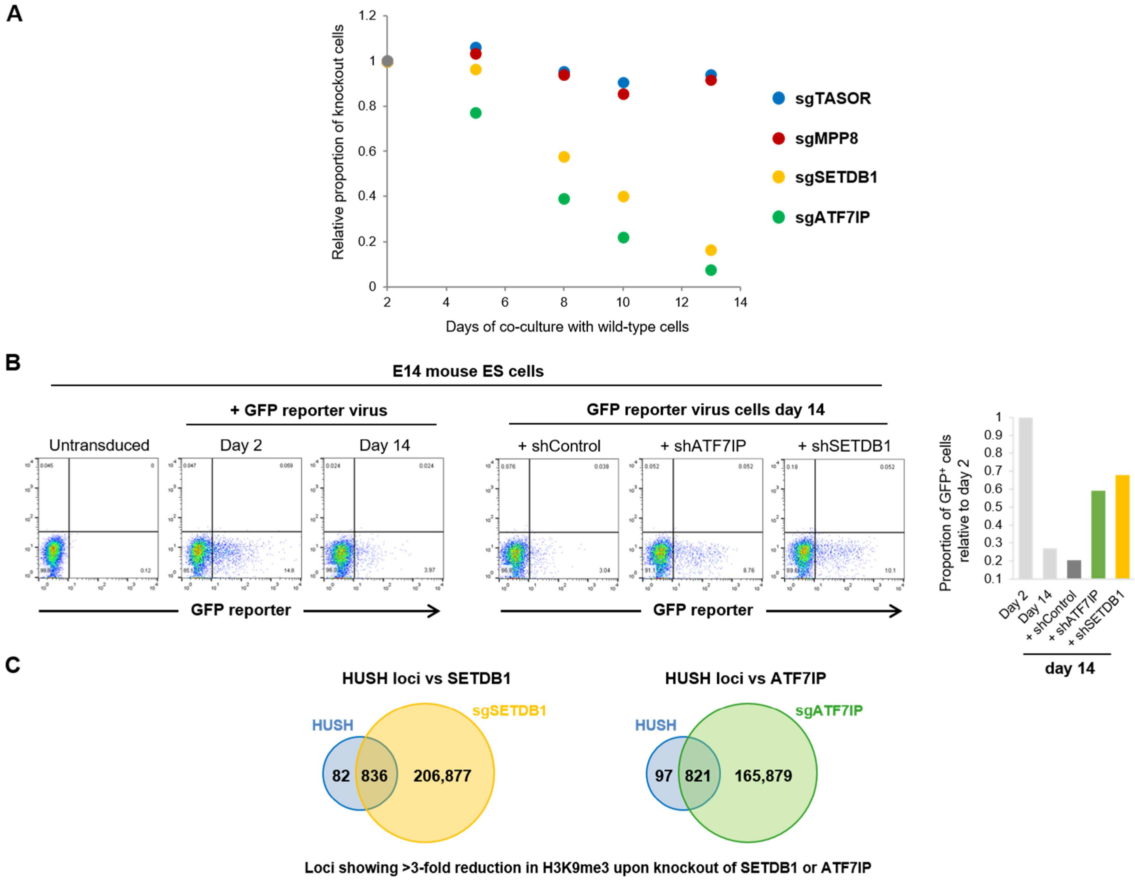

Figure S3. Loss of ATF7IP has similar phenotypic consequences to loss of SETDB1, Related to Figures 3 and 4.

(**A**) Ablation of ATF7IP impairs the growth of HeLa cells to a similar extent to ablation of SETBD1. Wild-type HeLa cells were mixed together with an approximately equal proportion of either ATF7IP or SETDB1 knockout reporter cells. As the knockout cells are GFP^{+} owing to derepression of the GFP reporter, dilution of the knockout cells from the population could be followed by flow cytometry. In contrast, cells lacking the HUSH subunits TASOR and MPP8 did not display a significant growth defect compared to the parental wild-type cells.

(**B**) Knockdown of ATF7IP impairs the repression of exogenous viral constructs in murine ES cells to a similar extent to knockdown of SETDB1. mESCs were transduced with a lentiviral vector encoding GFP, which was progressively silenced over the course of a few days. Reactivation of the GFP reporter did not occur upon transduction with a control shRNA, but an increase in the proportion of GFP+ cells was observed upon transduction with shRNAs targeting either SETDB1 or ATF7IP.

(**C**) SETDB1 and ATF7IP are responsible for H3K9me3 deposition at the vast majority of genomic loci at which the HUSH complex acts. Previously we identified 918 genomic loci at which knockout of either TASOR, MPP8 or Periphilin resulted in a significant decrease in H3K9me3 levels (Tchasovnikarova et al., 2015): 836 of these loci also exhibit a >3-fold reduction in H3K9me3 upon knockout of SETDB1, as do 821 of these loci upon knockout of ATF7IP.

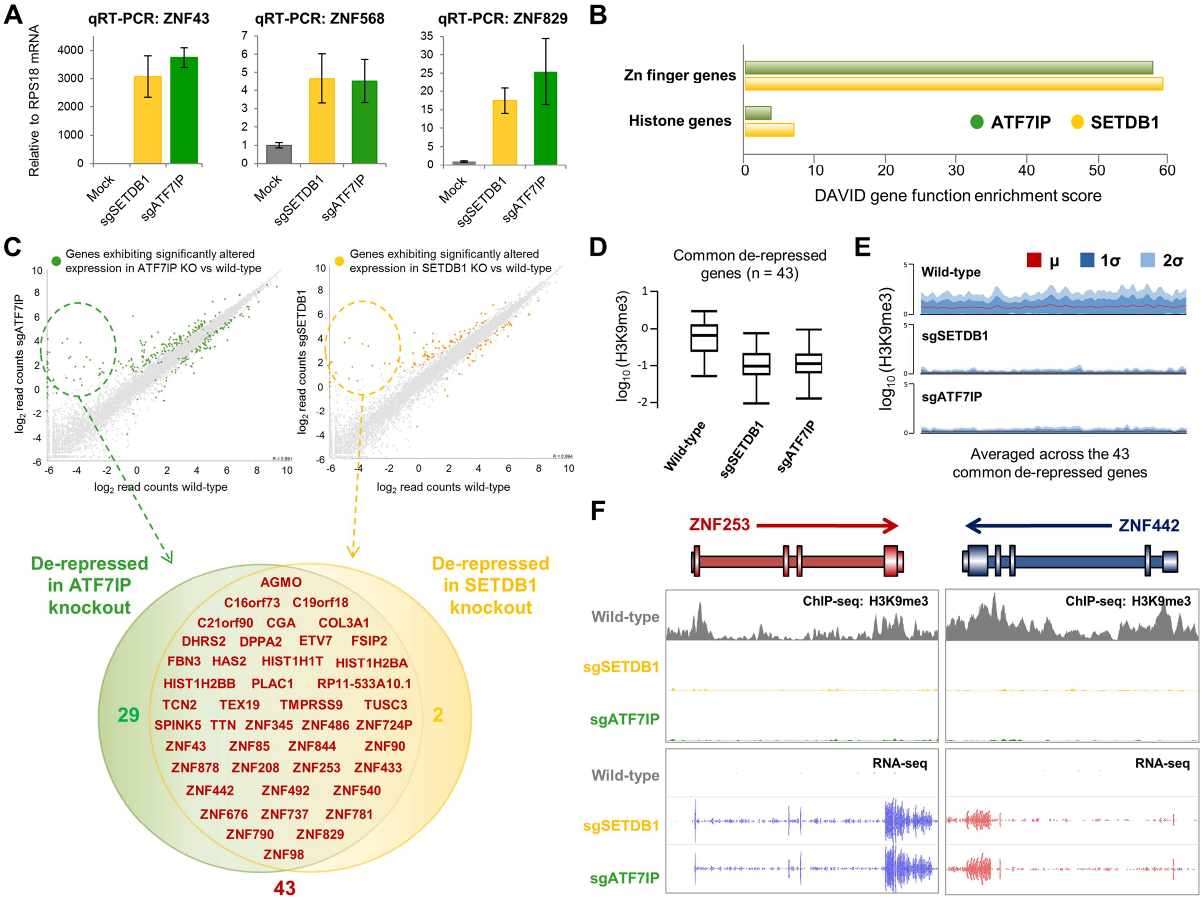

Figure S4. Loss of ATF7IP and SETDB1 has a similar impact on the transcriptome, Related to Figure 4.

(**A**) Validation of the RNA-seq data by qRT-PCR at three example KRAB-ZNF genes. Error bars represent the standard deviation of three qPCR technical replicates.

(**B**) Functional classification of the genes upregulated in ATF7IP and SETDB1 knockout cells using DAVID.

(**C**) Identification of genes that were lowly or not expressed in wild-type cells but which were significantly upregulated upon knockout of ATF7IP or SETDB1. This generated a common set of 43 derepressed genes.

(**D-F**) Transcriptional derepression upon loss of ATF7IP or SETDB1 results from a loss of H3K9me3 deposition. H3K9me3 levels across the common set of 43 upregulated genes were significantly reduced upon ATF7IP or SETDB1 knockout (D,E). H3K9me3 ChIP-seq and RNA-seq traces for two example zinc finger genes are shown in (F).

SUPPLEMENTAL TABLES

Table S1. Putative SETDB1-interacting proteins identified by mass spectrometry, Related to Figure 1.

Uniprot Accession	Protein	Number of peptides: Wild-type	Number of peptides: SETDB1 KO	Mw (kDa)
G3V1U0	Activating transcription factor 7 interacting protein (ATF7IP)	11	0	136.23
O00160	Unconventional myosin-If (MYO1F)	10	0	124.77
Q96C19	EF-hand domain-containing protein D2 (EFHD2)	6	0	26.68
P07195	L-lactate dehydrogenase B chain (LDHB)	4	0	36.61
Q86T24	Transcriptional regulator Kaiso (ZBTB33)	3	0	74.43

Table S2. Oligonucleotide sequences, Related to all Figures.

**sgRNAs**	**20 bp targeting sequence (5'-3')**
sg1-ATF7IP	GAGGCAGCATCACTAGAGGA
sg2-ATF7IP	ACTAGAGGCTATATCATCAG
sg3-ATF7IP	GGATGGCTCTGTAGAAGCTG
sg1-SETDB1	TGGATGCATCATCAAAGAGT
sg2-SETDB1	AACTCTTTGATGATGCATCC
sg3-SETDB1	GCTCTGAGGACGAATCTTCC
sg4-SETDB1	CAGAACTCCAAAAGACCAGA
**shRNAs**	**Forward oligonucleotide sequence (5'-3')**
shControl	GATCCGGGTATCGACGATTACAAATTCAAGAGATTTGTAATCGTCGATACCCTTTTTTG
sh1-ATF7IP	GATCCGGAAGAGGTGAATGGCATTTTCAAGAGAAATGCCATTCACCTCTTCCTTTTTTG
sh2-ATF7IP	GATCCGCCAAGATAGCCAGGTTAACTTCAAGAGAGTTAACCTGGCTATCTTGGTTTTTTG
shATF7IP (mouse)	GATCCGCGACGAACACTTAGACCAATTCAAGAGATTGGTCTAAGTGTTCGTCGTTTTTTG
shSETDB1 (mouse)	GATCCGAGGAACTTCGTCAGTACATTCAAGAGATGTACTGACGAAGTTCCTCTTTTTTG
**qPCR primers**	**Forward oligonucleotide sequence (5'-3')**
ATF7IP_F	ATGTGGTTGTTTGAGGCACT
ATF7IP_R	CCTTGCGAGGAACTGTTATG
RPS18_F	GCGGCGGAAAATAGCTTG
RPS18_R	TGAGTTCTCCCGCCCTCTTG
ZNF37A_F	CATTCTGAAGAGGAACCTTCTG
ZNF37A_R	TTTCTGATGCCAAATGAGGT
ZNF221_F	AAGGCATGATTTCACCTTCA
ZNF221_R	CTGCCTCTTTGAATGTGGTC
ZNF594_F	GCCTGGAGATTCTGAAGACA
ZNF594_R	CACATTCCTGGGTGATTTGT
ZNF43_F	GACCCTCATACCTGGAACCT
ZNF43_R	GTGCAGGCTGAAGAAGAGTC
ZNF568_F	CAGGCCTCTTGAAACAGTGA
ZNF568_R	TTTGAGCAGGTTTCATTTGC
ZNF829_F	GAGGATACCGAAGACCGATG
ZNF829_R	AGAGAGGTTTCCATGTTGGC

SUPPLEMENTAL EXPERIMENTAL PROCEDURES

Cell culture. HeLa cells were obtained from the ECACC and were grown in RPMI 1640 plus 10% FCS and penicillin/streptomycin, and, where stated, treated with the proteasome inhibitor Bortezomib (NEB) at a final concentration of 20 nM for 40 h. HEK 293ET cells, a generous gift from Dr. Felix Randow (MRC-LMB, Cambridge, UK), were cultured in IMDM plus 10% fetal calf serum (FCS) and penicillin/streptomycin. E14 mouse embryonic stem cells were a generous gift from Prof. Berthold Göttgens (CIMR, Cambridge, UK) and were cultured on gelatinized plates in KnockOut DMEM (Thermo Fisher Scientific) supplemented with 15% fetal bovine serum, 1000 U/ml LIF (Millipore), 2 mM L-glutamine, 0.1 mM β-mercaptoethanol and penicillin/streptomycin.

Antibodies. Primary antibodies used were as follows: rabbit α-SETDB1 (Proteintech, 11231-1-AP, 1:5000), rabbit α-ATF7IP (Bethyl Laboratories, A300-169A, 1:1000), rabbit α-GAPDH (Cell Signaling, #2118, 1:5000), mouse α-α-tubulin (Cell Signaling, #3873, 1:5000), rabbit α-HP1α (Cell Signaling, #2616, 1:5000), mouse α-β-actin (Sigma-Aldrich, A5316; 1:10000) and goat α-lamin B1 (SantaCruz Biotech, sc-20682, 1:1000).

CRISPR/Cas9-mediated gene disruption. Oligonucleotides (obtained from Sigma-Aldrich) encoding the for top and bottom strands of the sgRNA were phosphorylated using T4 polynucleotide kinase (NEB), annealed by heating to 95°C followed by slow cooling to room temperature, and then cloned into either the dual Cas9/sgRNA expression vector pSpCas9(BB)-2A-Puro (Addgene #48139, deposited by Dr. Feng Zhang (Ran et al., 2013)) or the lentiviral sgRNA expression vector pKLV-U6gRNA(BbsI)-PGKpuro2ABFP (Addgene

#50946, deposited by Dr. Kosuke Yusa (Koike-Yusa et al., 2014)). The sgRNAs expressed from pSpCas9(BB)-2A-Puro were transfected into HeLa cells using TransIT-HeLaMONSTER (Mirus) according to the manufacturer's protocol, and the transfected cells enriched by two days of puromycin selection starting 24 hours after transfection. For lentiviral CRISPR/Cas9-mediated gene disruption, cells were first transduced with the Cas9 expression vector pHRSIN-P_{SFFV}-Cas9-P_{PGK}-Hygro, selected on hygromycin, and then subsequently transduced with the lentiviral sgRNA expression vector and selected using puromycin. All sgRNA sequences are detailed in Table S2.

RNA interference. Lentiviral expression of shRNA constructs was achieved using the pHR-SIREN vector, with hairpins cloned in as BamHI-EcoRI fragments. All shRNA sequences are detailed in Table S2.

Lentiviral expression. Lentivirus was produced through the triple transfection of HEK 293ET cells with three plasmids: the lentiviral transfer vector, and the packaging plasmids pCMVΔR8.91 and pMD.G. Transfection was achieved using the TransIT-293 reagent (Mirus) as recommended by the manufacturer. The viral supernatant was collected 48 h later, passed through a 0.45 μm filter, and target cells transduced by spin infection at 700 x *g* for 60 min. The viral vector used to transduce mouse ES cells was a pHRSIN-based lentivirus encoding GFP from the Rous sarcoma virus (RSV) LTR promoter (pHRSIN-P_{RSV}-GFP).

Flow cytometry. Harvested cells were washed once with PBS and then fixed in 1% formaldehyde prior to analysis on a FACSCalibur instrument (BD). Cell sorting was carried out on an Influx cell sorter (BD).

Immunoblotting. Cells were lysed in 1% SDS in TBS plus protease inhibitor cocktail (Roche) at room temperature for 30 min. Samples were then heated in SDS sample buffer for 10 min at 70°C, separated by SDS-PAGE, and transferred to a PVDF membrane (Millipore). The membranes were blocked by incubation in 5% milk in PBS + 0.2% Tween-20 for at least 30 min, and then probed overnight at 4°C with the indicated primary antibodies. Following 3 x 5 min washes with PBS + 0.2% Tween-20, membranes were incubated with HRP-conjugated secondary antibodies for 45 min at room temperature and reactive bands visualized using ECL western blotting substrate, West Pico or West Dura (Thermo Fisher Scientific).

Quantitative reverse transcription PCR (qRT-PCR). RNA was extracted from HeLa cells with the RNeasy Plus kit (Qiagen) and then converted into cDNA using Super RT reverse transcriptase (HT Biotechnology) with a poly(d)T primer following the manufacturer's instructions. Samples were analyzed on an ABI 7500 Real Time PCR System (Applied Biosystems) using SYBR green PCR mastermix (Life Technologies). PCR reactions were performed in a total reaction volume of 25 µl, using 20 ng of cDNA, 10 µl of SYBR green PCR mastermix and 0.2 µM of forward and reverse primers. Cycling parameters were 50°C for 2 min and 95°C for 5 min, with a subsequent 40 cycles of 95°C for 15 s, 58°C for 1 min. Primer sequences are detailed in Table S2.

Statistical methods. All qPCR data is represented as the mean +/- standard deviation of three technical replicates. For the ChIP-qPCR data presented in Figure 3D, individual dots represent 1 kb genomic intervals; only intervals with greater than 40 reads in one of the samples were used for calculating the Pearson correlation coefficient. For the RNA-seq data presented in Figure 4B-E, the highlighted genes exhibited differential expression as determined by DEseq ($P < 0.05$) and also passed the Intensity Difference filter in SeqMonk.

SUPPLEMENTAL REFERENCES

Koike-Yusa, H., Li, Y., Tan, E.-P., Velasco-Herrera, M.D.C. and Yusa, K. (2014). Genome-wide recessive genetic screening in mammalian cells with a lentiviral CRISPR-guide RNA library. Nat. Biotechnol. *32*, 267–73.

Ran, F.A., Hsu, P.D., Wright, J., Agarwala, V., Scott, D.A. and Zhang, F. (2013). Genome engineering using the CRISPR-Cas9 system. Nat. Protoc. *8*, 2281–308.
