# Supplementary material for: ATF7IP-Mediated Stabilization of the Histone Methyltransferase SETDB1 Is Essential for Heterochromatin Formation by the HUSH Complex
Source: Cell Rep. 2016 Oct 11;17(3):653–9. doi: 10.1016/j.celrep.2016.09.050 (PMC5081395; doi:10.1016/j.celrep.2016.09.050)
Supplement: Document S2. Article plus Supplemental Information [file mmc2.pdf]

Report

Cell Reports

ATF7IP-Mediated Stabilization of the Histone Methyltransferase SETDB1 Is Essential for Heterochromatin Formation by the HUSH Complex

Graphical Abstract

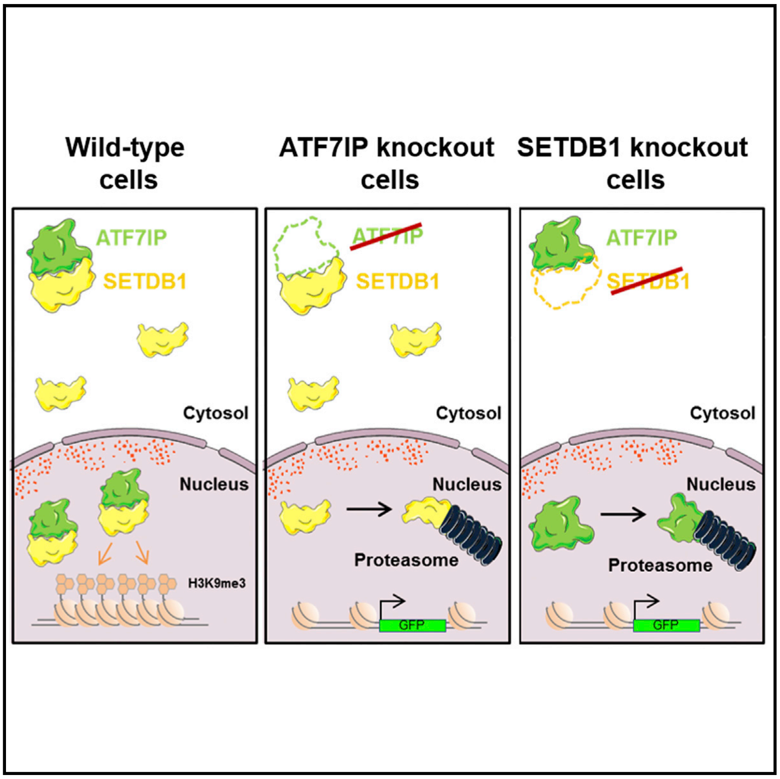

Highlights

- The SETDB1-interacting partner ATF7IP is critical for HUSH-mediated silencing
- ATF7IP shields SETDB1 from proteasomal degradation in the nucleus
- Loss of ATF7IP phenocopies loss of SETDB1 in genome-wide assays

Authors

Richard T. Timms, Iva A. Tchasovnikarova, Robin Antrobus, Gordon Dougan, Paul J. Lehner

Correspondence

pjl30@cam.ac.uk

In Brief

The histone lysine methyltransferase SETDB1 is a central chromatin regulator, but the function of its binding partner ATF7IP has remained enigmatic. Timms et al. find that ATF7IP plays an essential role in heterochromatin formation by shielding SETDB1 from proteasomal degradation in the nucleus.

Accession Numbers

GSE86814

Timms et al., 2016, Cell Reports *17*, 653–659
October 11, 2016 © 2016 The Author(s).
http://dx.doi.org/10.1016/j.celrep.2016.09.050

Cell Reports
Report

OPEN ACCESS
CellPress

ATF7IP-Mediated Stabilization of the Histone Methyltransferase SETDB1 Is Essential for Heterochromatin Formation by the HUSH Complex

Richard T. Timms,[1,3] Iva A. Tchasovnikarova,[1,3] Robin Antrobus,[1] Gordon Dougan,[2] and Paul J. Lehner[1,4,*]
[1]Department of Medicine, Cambridge Institute for Medical Research, Cambridge Biomedical Campus, Cambridge CB2 0XY, UK
[2]Wellcome Trust Sanger Institute, Wellcome Trust Genome Campus, Cambridge CB10 1SA, UK
[3]Co-first author
[4]Lead Contact
*Correspondence: pjl30@cam.ac.uk
http://dx.doi.org/10.1016/j.celrep.2016.09.050

SUMMARY

The histone methyltransferase SETDB1 plays a central role in repressive chromatin processes, but the functional requirement for its binding partner ATF7IP has remained enigmatic. Here, we show that ATF7IP is essential for SETDB1 stability: nuclear SETDB1 protein is degraded by the proteasome upon ablation of ATF7IP. As a result, ATF7IP is critical for repression that requires H3K9 trimethylation by SETDB1, including transgene silencing by the HUSH complex. Furthermore, we show that loss of ATF7IP phenocopies loss of SETDB1 in genome-wide assays. ATF7IP and SETDB1 knockout cells exhibit near-identical defects in the global deposition of H3K9me3, which results in similar dysregulation of the transcriptome. Overall, these data identify a critical functional role for ATF7IP in heterochromatin formation by regulating SETDB1 abundance in the nucleus.

INTRODUCTION

The post-translational modification of histone proteins is a critical mechanism to control chromatin architecture and gene expression in eukaryotic cells (Kouzarides, 2007). SETDB1 (also known as ESET) is a key enzyme responsible for methylation of lysine 9 of histone H3 (H3K9me), which is a hallmark of repressed chromatin (Becker et al., 2016). However, four additional methyltransferases (G9a, GLP, SUV39H1, and SUV39H2) are also known to target H3K9, raising the question as to how the activities of these enzymes are coordinately regulated to generate the required distribution of H3K9 methylation across the genome (Mozzetta et al., 2015). The importance of correct regulation of SETDB1 is underscored by the oncogenic potential of SETDB1 overexpression, with amplification of SETDB1 implicated in the progression of malignant melanoma (Ceol et al., 2011) and cancers of the prostate (Sun et al., 2014), liver (Fei et al., 2015; Wong et al., 2016), and lung (Rodriguez-Paredes et al., 2014; Sun et al., 2015).

Binding partners provide one mechanism to regulate the activity of these methyltransferases. ATF7IP (also known as MCAF1 or hAM) has been recognized as a SETDB1-interacting protein for more than a decade (Fujita et al., 2003; Wang et al., 2003), but its functional role has remained elusive. Initially, ATF7IP was proposed to act as a direct modulator of the catalytic activity of SETDB1, with mAM, the murine homolog of ATF7IP, shown to stimulate the catalytic activity of murine SETDB1 in vitro and promote the efficient conversion of di-methyl H3K9 to the tri-methylated state (Wang et al., 2003). However, a recent study found little effect of human ATF7IP on the in vitro catalytic activity of human SETDB1 (Basavapathruni et al., 2016), leading to the suggestion that the in vivo role of ATF7IP could be to regulate the spatial or temporal recruitment of SETDB1 to target sites on chromatin (Basavapathruni et al., 2016; Koch et al., 2009).

Previously, we identified the Human Silencing Hub (HUSH) complex as a regulator of heterochromatin formation in mammalian cells (Tchasovnikarova et al., 2015). HUSH comprises three proteins, TASOR, MPP8, and Periphilin, which together recruit SETDB1 to deposit H3K9me3 and mediate transcriptional repression at heterochromatic loci (Tchasovnikarova et al., 2015). While searching for proteins required alongside SETDB1, we identified an essential role for ATF7IP in HUSH complex function. Here we clarify the function of ATF7IP, showing that it plays a critical role in SETDB1-mediated H3K9me3 deposition by shielding SETDB1 from proteasomal degradation in the nucleus.

RESULTS

The SETDB1-Interacting Partner ATF7IP Is Essential for Epigenetic Silencing by the HUSH Complex

We took a proteomic approach to identify proteins that act alongside SETDB1 in HUSH-mediated epigenetic silencing. Immunoprecipitation of SETDB1 from the nuclei of HeLa cells followed by mass spectrometry (Figure 1A) identified ATF7IP as the top candidate that was not present in the control sample (Figure 1B; Table S1). We were readily able to validate the interaction between SETDB1 and ATF7IP in a reciprocal co-immunoprecipitation experiment (Figure 1C), a finding that is consistent with a number of previous reports (Ichimura et al., 2005; Koch et al., 2009; Wang et al., 2003). Because H3K9me3 deposition

This is an open access article under the CC BY license (http://creativecommons.org/licenses/by/4.0/).

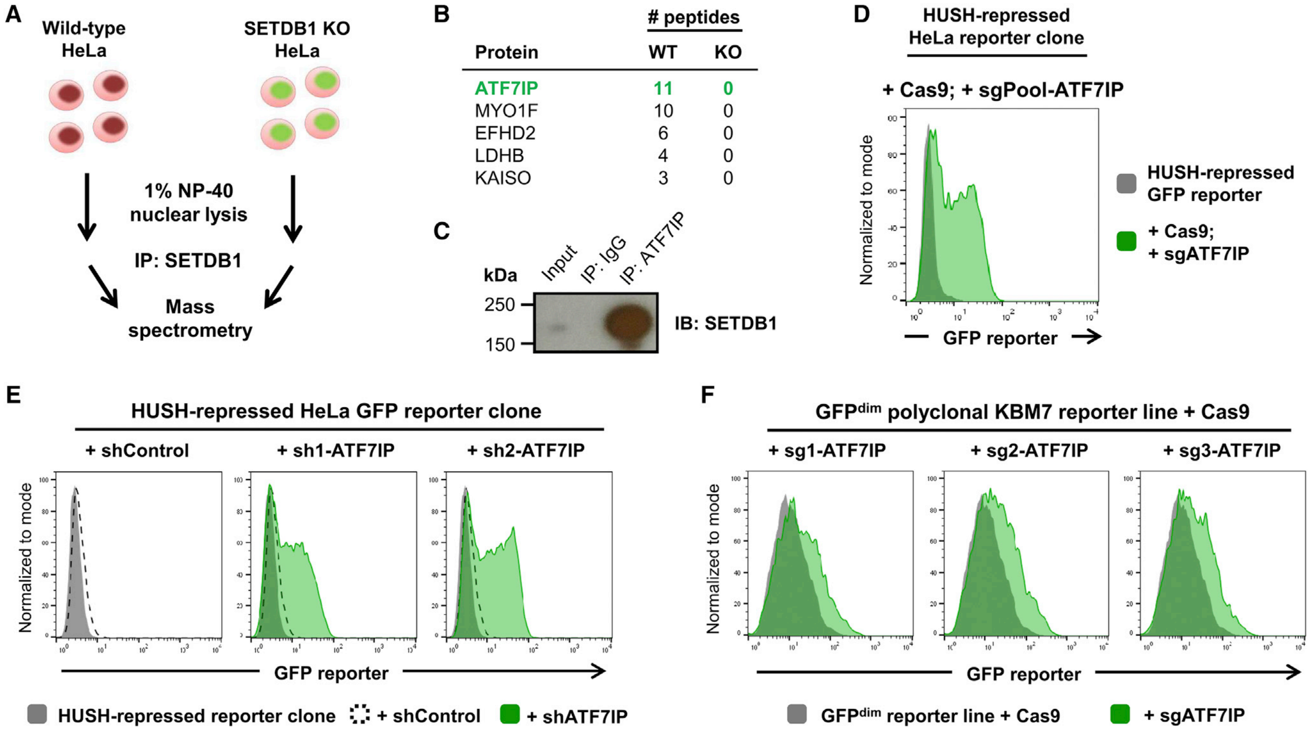

Figure 1. The SETDB1-Interacting Protein ATF7IP Is Critical for HUSH-Mediated Epigenetic Repression

(A–C) SETDB1 binds ATF7IP. Immunoprecipitation of SETDB1 from HeLa nuclear lysates followed by mass spectrometry (A) identified ATF7IP as the most significantly enriched co-immunoprecipitating protein (B); immunoprecipitation of ATF7IP also pulled down SETDB1 (C).

(D) ATF7IP is critical for transgene silencing by the HUSH complex. CRISPR/Cas9-mediated disruption of ATF7IP resulted in the reactivation of a HUSH-repressed GFP reporter.

(E) Depletion of ATF7IP results in reactivation of a silent GFP reporter construct. A HeLa clone harboring a HUSH-repressed GFP reporter was transduced with lentiviral vectors expressing either a control shRNA or shRNAs targeting ATF7IP, and derepression of the GFP reporter construct was assayed by flow cytometry.

(F) Knockout of ATF7IP results in derepression of HUSH-repressed GFP reporter constructs in KBM7 cells. A polyclonal population of KBM7 cells harboring HUSH-repressed GFP reporter constructs was transduced sequentially with lentiviral vectors expressing Cas9 and single guide RNAs (sgRNAs) targeting ATF7IP, and depression of the GFP reporter constructs was monitored by flow cytometry.

See also Figures S1 and S2.

by SETDB1 is critical for HUSH-mediated transgene repression (Tchasovnikarova et al., 2015), we examined whether ATF7IP was also essential for this process. We ablated ATF7IP function in a HeLa clone harboring a HUSH-repressed GFP reporter construct (Tchasovnikarova et al., 2015), through either short hairpin RNA (shRNA)-mediated knockdown or CRISPR/Cas9-mediated gene disruption, and examined the impact on reporter silencing by flow cytometry. In both cases, loss of ATF7IP resulted in the abrogation of HUSH-mediated silencing and derepression of the reporter construct (Figures 1D and 1E). Similar results were observed in a polyclonal KBM7 reporter line harboring HUSH-repressed GFP transgenes (Figure 1F). Therefore, ATF7IP is critical for HUSH-mediated transgene silencing.

ATF7IP Protects SETDB1 from Proteasomal Degradation in the Nucleus

To characterize the functional role of ATF7IP, we generated ATF7IP-deficient HeLa clones through CRISPR/Cas9-mediated gene disruption (Figures S1A and S1B). Our antibody against ATF7IP was not capable of recognizing its epitope in direct immunoblot analysis (Figure S1C), but we were able to validate an absence of ATF7IP protein by immunoprecipitation of ATF7IP followed by immunoblot (Figure S1D). The overall abundance of SETDB1 was not significantly affected in the ATF7IP knockout clones (Figure 2A), but, strikingly, SETDB1 was lost from the nucleus in the absence of ATF7IP (Figure 2B). We observed a similar outcome following shRNA-mediated depletion of ATF7IP (Figures S1E and S1F). This result suggested two potential roles for ATF7IP that are not mutually exclusive: import of SETDB1 into the nucleus and stabilization of SETDB1 in the nucleus. Treatment of ATF7IP knockout cells with the proteasome inhibitor bortezomib (Velcade) resulted in marked recovery of SETDB1 protein levels in the nucleus (Figure 2C), demonstrating that SETDB1 was able to enter the nucleus in the absence of ATF7IP but in doing so became a substrate for proteasomal degradation (Figure 2C). Therefore, ATF7IP stabilizes SETDB1 in the nucleus.

We also examined the fate of ATF7IP in the absence of SETDB1. In SETDB1 knockout clones generated through CRISPR/Cas9-mediated gene disruption (Figure S2), the levels of ATF7IP protein were severely reduced (Figure 2D). In wild-type cells, ATF7IP was present almost exclusively in the nuclear

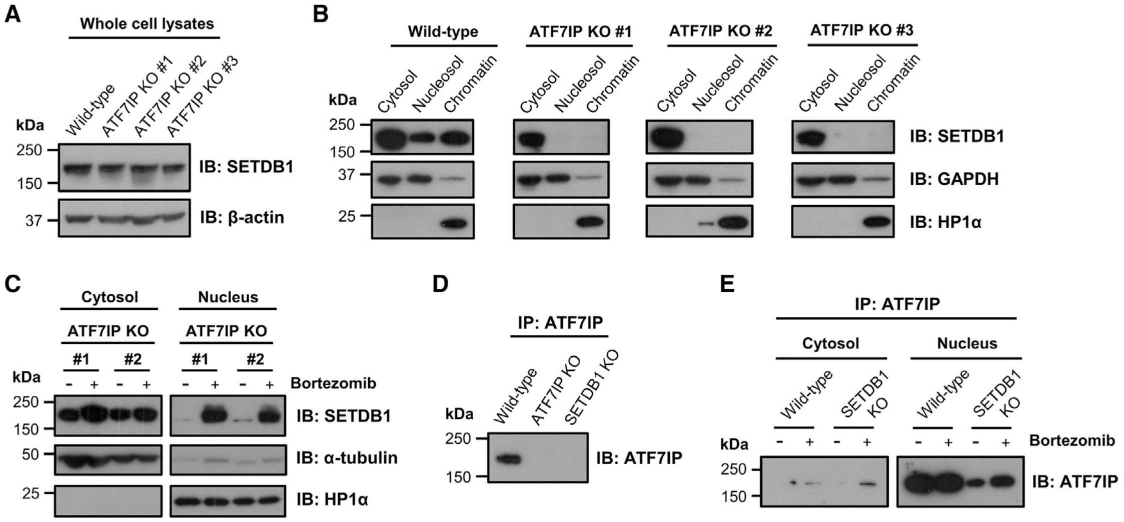

Figure 2. ATF7IP Shields SETDB1 from Proteasomal Degradation in the Nucleus

(A and B) Loss of ATF7IP abolishes the nuclear pool of SETDB1. The overall abundance of SETDB1 protein was not significantly affected in three independent ATF7IP knockout clones as assessed by immunoblot (A), but subcellular fractionation revealed a loss of SETDB1 from the nuclear fractions (B).

(C) ATF7IP protects SETDB1 from proteasomal degradation in the nucleus. Treatment of ATF7IP knockout cells with the proteasome inhibitor bortezomib (20 nM for 40 hr) recovered SETDB1 levels in the nuclear fraction.

(D and E) ATF7IP is destabilized in the absence of SETDB1. Loss of SETDB1 results in destabilization of ATF7IP (D), which can be recovered upon treatment with bortezomib (E).

See also Figure S2.

fraction, and bortezomib treatment of SETDB1 knockout cells was able to partially rescue nuclear ATF7IP levels (Figure 2E). Therefore, ATF7IP and nuclear SETDB1 are responsible for each other's stability.

Loss of ATF7IP Phenocopies that of SETDB1 Deletion

This reciprocal relationship between SETDB1 and ATF7IP predicts that the loss of ATF7IP should phenocopy that of SETDB1 deletion. We tested this hypothesis in a range of experimental systems. First, using competitive growth assays, we demonstrated that knockout of ATF7IP and SETDB1 had a similarly detrimental effect on the growth of HeLa cells (Figure S3A). In contrast to HeLa cells lacking the HUSH components TASOR or MPP8, cells lacking either ATF7IP or SETDB1 (as marked by GFP reporter expression) were progressively diluted from the population upon co-culture with the parental wild-type cells (Figure S3A).

In murine embryonic stem cells (mESCs), retroelements are epigenetically repressed through H3K9me3 in a process involving the KAP1-dependent recruitment of SETDB1 (Matsui et al., 2010; Rowe et al., 2010). We found that, as observed upon depletion of SETDB1, shRNA-mediated knockdown of ATF7IP in mESCs resulted in depression of an exogenous viral GFP reporter (Figure S3B). This finding is consistent with the results of a genome-wide screen to identify viral silencing factors in mESCs, which was published during the preparation of this manuscript (Yang et al., 2015).

Previously, we showed that SETDB1 deletion in HeLa cells results in genome-wide loss of H3K9me3 across thousands of loci (Tchasovnikarova et al., 2015). To determine whether this effect was mirrored upon disruption of ATF7IP, we used chromatin immunoprecipitation followed by deep sequencing (ChIP-seq) to compare the distribution of H3K9me3 in SETDB1-null and ATF7IP-null clones. The distribution of H3K9me3 was almost identical in each case, with dramatic changes in H3K9me3 levels observed across large segments of the genome (Figures 3A–3C). Considering the genome as a series of 1 kb windows, this experiment identified 207,713 loci showing a >3-fold decrease in H3K9me3 levels in SETDB1 knockout cells when compared to the wild-type parental cell line; of these loci, 99.3% also showed a decrease in H3K9me3 in ATF7IP knockout cells. Similarly, of the 166,700 loci exhibiting a >3-fold loss of H3K9me3 in ATF7IP knockout cells, 99.7% displayed a decrease in H3K9me3 levels in SETDB1 knockout cells. The overall concordance between the two knockout samples was extremely high (Spearman's rank correlation coefficient $r = 0.88$) (Figure 3D), and we validated these findings at several example loci by ChIP-qPCR in an independent experiment (Figure 3E). Previously we identified 918 genomic loci at which knockout of either of the HUSH subunits TASOR, MPP8, or Periphilin resulted in a significant decrease in H3K9me3 (Tchasovnikarova et al., 2015); at 98% of these loci, we also observed a decrease in H3K9me3 upon knockout of either SETDB1 or ATF7IP, with ~90% of the loci in each case exhibiting a decrease >3-fold (Figure S3C). One of the most striking effects of SETDB1 or ATF7IP depletion was loss of H3K9me3 across genes encoding zinc-finger proteins (Figures 3C and 3F). The deposition of H3K9me3 across the bodies of these genes is dependent on the function of the SETDB1-ATF7IP complex (Figure 3G), with the magnitude of the effect upon knockout of either component showing high concordance (Figure 3H).

Finally, we compared the global effects of deletion of SETDB1 and ATF7IP on the transcriptome by carrying out RNA

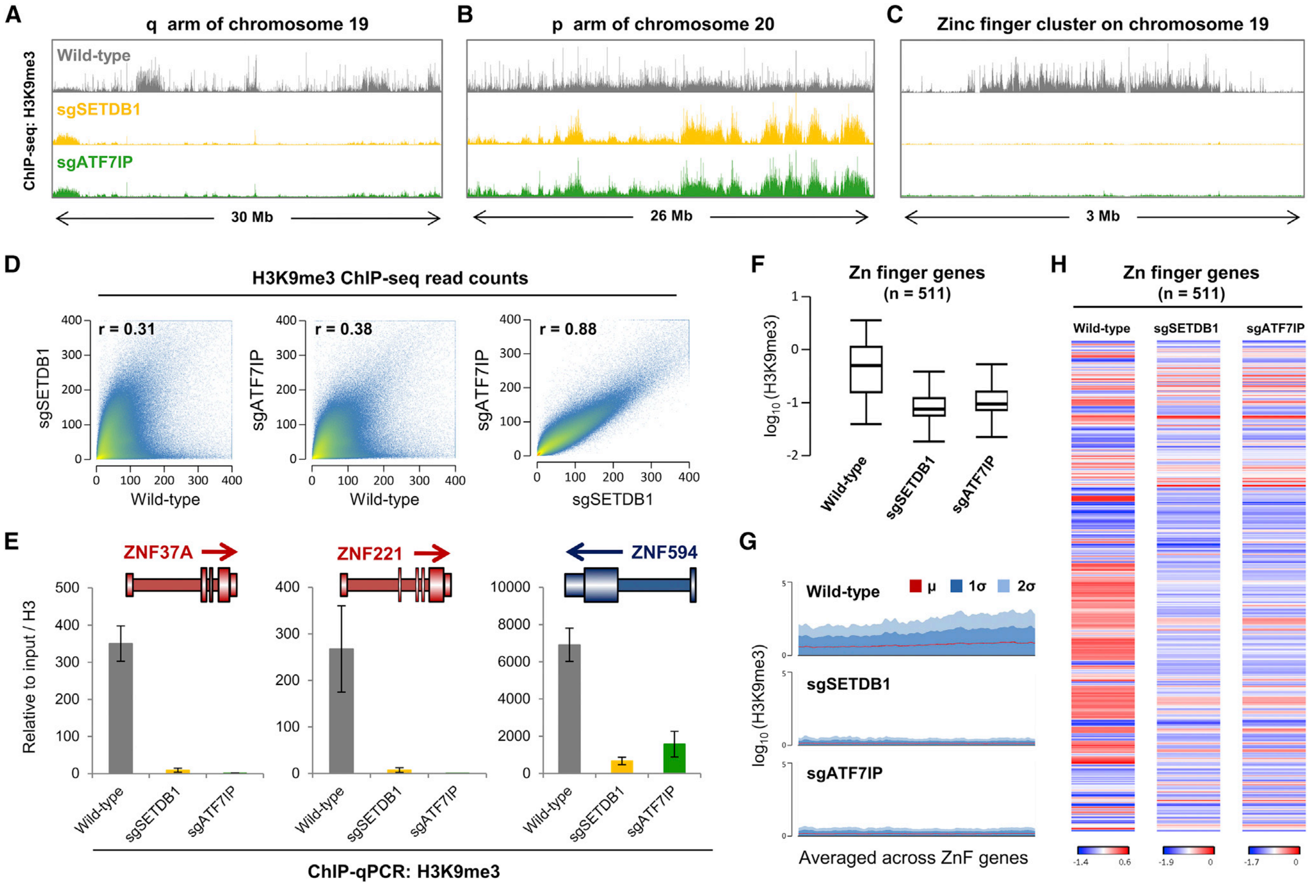

Figure 3. H3K9me3 Loss across the Genome Is Similar upon Deletion of ATF7IP or SETDB1

(A–D) The distribution of H3K9me3 in wild-type, ATF7IP knockout, and SETDB1 knockout cells was assessed by ChIP-seq analysis. Three example loci are shown (A–C); near-identical profiles were observed in the ATF7IP and SETDB1 knockout cells (D).

(E–H) Loss of ATF7IP and SETDB1 results in loss of H3K9me3 across zinc-finger genes. Validation of the ChIP-seq results by ChIP-qPCR at three example KRAB-ZNF gene loci (E). A near-total loss of H3K9me3 was observed across zinc-finger genes in the knockout cells (F and G), and the magnitude of this effect was similar in the ATF7IP and SETDB1 knockout samples (H). The error bars in (E) represent the SD of three qPCR technical replicates.

See also Figures S3 and S4.

sequencing (RNA-seq) analysis in the knockout clones (Figure 4A). Depletion of either SETDB1 or ATF7IP resulted in significantly altered expression of >1,000 genes in each case compared to wild-type cells (Figures 4B, 4C, and S4A). However, only a handful of genes ($n = 9$, $p < 0.05$ DEseq) exhibited differential expression when comparing the transcriptome of SETDB1 knockout cells to that of ATF7IP knockout cells (Figures 4D and 4E). Of particular interest were the genes upregulated in SETDB1 and ATF7IP knockout cells compared to wild-type cells. The DAVID functional annotation tool (Huang et al., 2009) identified two classes of derepressed genes in each case: zinc-finger genes and histone genes (Figure S4B). We focused on those genes that were either not expressed or weakly expressed in wild-type cells but became strongly expressed in the knockout cells; this generated a common set of 43 genes (Figure S4C). In support of the idea that the increased expression of these genes was due to loss of H3K9 methylation in the absence of SETDB1 and ATF7IP, H3K9me3 levels were substantially reduced across these genes in both sets of knockout cells (Figures S4D and S4E), concomitant with the increase in transcription (Figure S4F). Altogether, both the ChIP-seq and the RNA-seq datasets supported a functional co-dependence between SETDB1 and ATF7IP.

DISCUSSION

The goal of this study was to clarify the functional role of the SETDB1-binding partner ATF7IP. We found that in the absence of ATF7IP, SETDB1 was not stable in the nucleus and was subject to proteasomal degradation. Overall, these data explain why ATF7IP is critical for transgene silencing by the HUSH complex and other repressive epigenetic processes that require H3K9me3 deposition by SETDB1.

Although stabilization of SETDB1 is clearly a central function for ATF7IP, it seems unlikely that this is its only role. For example, the C-terminal fibronectin type-III domain of ATF7IP has been shown to bind the methyl-DNA binding protein MBD1 (Ichimura et al., 2005), with functional roles for MBD1 and ATF7IP identified

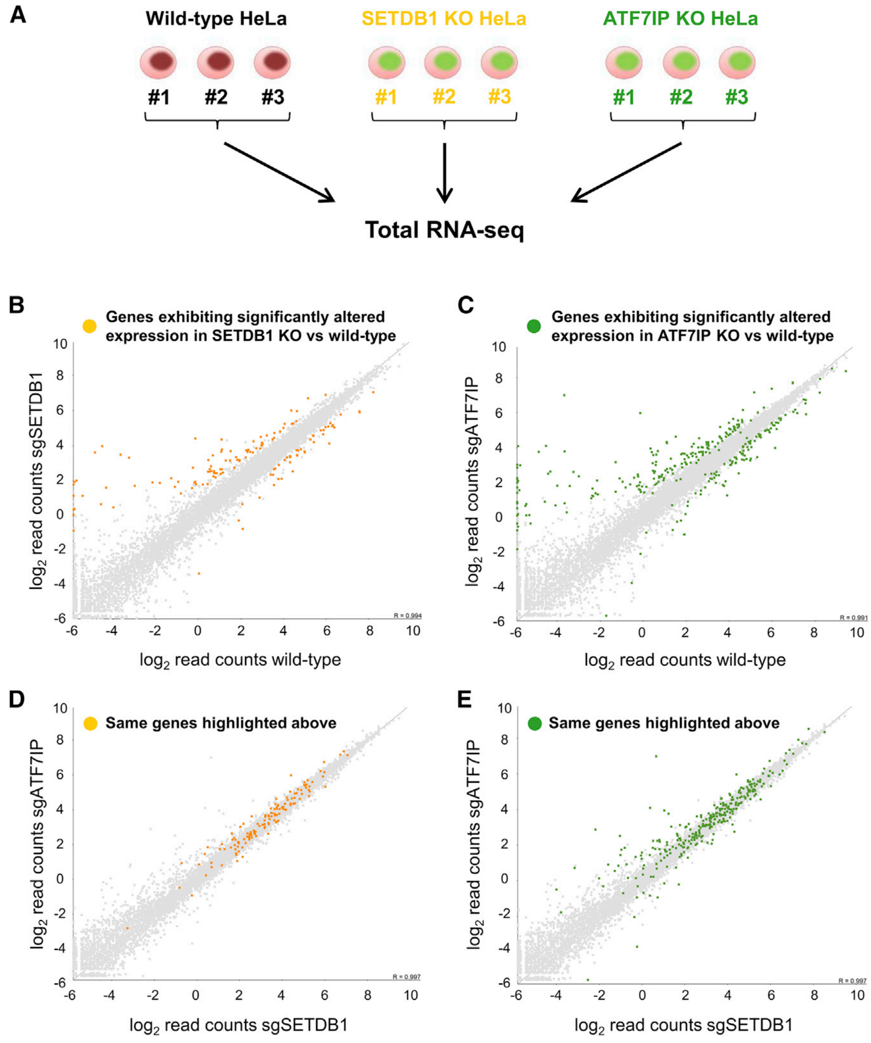

Figure 4. Deletion of ATF7IP Mirrors the Effect of SETDB1 Deletion on the Transcriptome

(A) Schematic representation of the RNA-seq experiment.

(B–E) Loss of ATF7IP and SETDB1 results in highly similar effects on the transcriptome. The colored dots represent genes exhibiting significantly altered expression (see Experimental Procedures) in ATF7IP knockout cells (green) or SETDB1 knockout cells (yellow) compared to wild-type cells (B and C); these genes are affected similarly in both knockout cells, because they lie in the center of the distribution when comparing ATF7IP knockout cells to SETDB1 knockout cells (D and E).

See also Figure S4.

in transcriptional regulation (Waterfield et al., 2014) and X chromosome inactivation (Minkovsky et al., 2014). It remains to be tested whether MBD1 also plays a critical role alongside SETDB1 and ATF7IP in HUSH-mediated epigenetic silencing.

SETDB1 does not appear to be an intrinsically unstable protein, given that it can be recombinantly expressed in isolation in insect cells (Wang et al., 2003) and is found in the cytosol in the absence of ATF7IP (Figure 2B). Therefore, it seems likely that an active degradation process removes unshielded SETDB1 in the nucleus, and an important goal for future work will be to delineate the degradative machinery responsible. However, it remains unclear what evolutionary advantage ATF7IP-mediated regulation of SETDB1 may provide. One possibility is that modulation of ATF7IP levels can provide dynamic regulation of SETDB1 methyltransferase activity during development or in response to specific stimuli. Alternatively, ATF7IP could simply act as a safety mechanism to prevent SETDB1 overactivity. Amplification of SETDB1 is heavily implicated in tumorigenesis (Ceol et al., 2011; Fei et al., 2015; Rodriguez-Paredes et al., 2014; Sun et al., 2014, 2015; Wong et al., 2016), and upregulation of ATF7IP has been observed in a number of tumors (Liu et al., 2009), suggesting that overexpression of SETDB1 could have deleterious effects. Given that no effective small molecule inhibitors of SETDB1 catalytic activity have been developed, our data raise the possibility that an alternative strategy to inhibit SETDB1 activity could be to target ATF7IP. Structural insight would be valuable in assessing the feasibility of targeting the SETDB1-ATF7IP interface for therapeutic benefit.

EXPERIMENTAL PROCEDURES

Co-immunoprecipitation and Mass Spectrometry

HeLa cells were grown in RPMI 1640 supplemented with 10% fetal calf serum and penicillin and streptomycin. Nuclei were extracted with 0.1% IGEPAL (Sigma-Aldrich) and then lysed in 1% IGEPAL plus 1:100 benzonase (Sigma-Aldrich). Nuclear lysates were pre-cleared with protein A and immunoglobulin G (IgG)-sepharose and then incubated with primary antibody and protein G-coated magnetic beads (Thermo Fisher Scientific) for 2 hr at 4°C. Following three washes in lysis buffer, bound proteins were eluted from the beads in SDS sample buffer. For analysis by mass spectrometry, immunoprecipitates were first resolved by SDS-PAGE, with each lane cut into four slices for in-gel digestion. Tryptic peptides were analyzed by liquid chromatography-tandem mass spectrometry (LC-MS/MS), and the raw data were processed in Proteome Discoverer v.1.4. Data were searched using Sequest against the Human Uniprot database (downloaded March 3, 2014; 68,710 sequences).

Subcellular Fractionation

One million HeLa cells were washed twice in PBS and once in Buffer A (10 mM HEPES, 1.5 mM $MgCl_2$, 10 mM KCl, 0.5 mM DTT, and an EDTA-free protease inhibitor cocktail tablet; Roche). Cells were then resuspended in Buffer A plus 0.1% (v/v) IGEPAL and incubated on ice for 10 min. The supernatant containing the cytosolic fraction was collected following centrifugation at 1,400 × *g* for 4 min at 4°C. To isolate the total nuclear fraction, the nuclear pellet was then lysed in 1% SDS plus 1:100 benzonase (Sigma-Aldrich) for 20 min at room temperature. For further separation into nucleosolic and chromatin fractions, the nuclear pellet was resuspended in Buffer B (20 mM HEPES, 1.5 mM

$MgCl_2$, 300 mM NaCl, 0.5 mM DTT, 25% v/v glycerol, 0.2 mM EDTA, and an EDTA-free protease inhibitor cocktail tablet) for 10 min on ice. Following centrifugation at 1,700 × *g* for 4 min at 4°C, the supernatant contained the nucleosolic fraction and the insoluble pellet the chromatin fraction. The pellet was then solubilized in 1% SDS plus 1:100 benzonase.

H3K9me3 ChIP-Seq

HeLa cells were washed once in PBS, resuspended in RPMI growth media, and then cross-linked for 10 min by the addition of 1% formaldehyde. The reaction was quenched for 5 min by the addition of glycine to a final concentration of 0.125 M, and the cells were lysed in cell lysis buffer (1 mM HEPES, 85 mM KCl, and 0.5% NP-40). The nuclei were then lysed in nuclear lysis buffer (5 mM Tris, 10 mM EDTA, and 1% SDS) and the chromatin was sheared using a Bioruptor (Diagenode; 20 cycles of 30 s on and 30 s off on high power) to obtain a mean fragment size of ~300 bp. The chromatin solution was then pre-cleared with protein A sepharose (Sigma-Aldrich), and the immunoprecipitation reaction was performed overnight using 5 µg of anti-H3K9me3 (Abcam; ab8898) primary antibody and protein A sepharose. The beads were washed five times before bound protein-DNA complexes were eluted with 150 mM $NaHCO_3$ and 1% SDS. Cross-links were reversed by the addition of 0.3 M NaCl and RNase A, followed by incubation at 67°C for 4 hr. Proteins were removed by the addition of Proteinase K for 2 hr at 45°C, and the DNA was purified using a spin column (QIAGEN PCR purification kit). Illumina sequencing libraries were created using the NEBNext ChIP-Seq Library Prep Kit (NEB) and sequenced on a HiSeq 2500 instrument. Reads were aligned to the human genome (GRCh37) using Bowtie2 and further analyzed using SeqMonk and EaSeq (Lerdrup et al., 2016).

RNA-Seq

RNA was extracted from three independent ATF7IP and SETDB1 knockout clones using the miRNEasy kit (QIAGEN) as recommended by the manufacturer. Genomic DNA was removed by on-column digestion with DNase I, and rRNAs were depleted from the resulting samples using the Ribo-Zero Gold rRNA Removal Kit (Epicenter). Multiplexed Illumina sequencing libraries were prepared using the TruSeq Stranded Total RNA Library Prep Kit (Illumina), and 150 bp paired-end reads were generated on a HiSeq 2500 instrument. Sequencing reads were aligned to the human genome (GRCh37) using HISAT2. Aligned reads with a MAPQ score > 40 were imported into SeqMonk and analyzed using the RNA-seq quantitation pipeline, followed by DEseq analysis. In Figures 4B–4E, the highlighted genes exhibited differential expression as determined by DEseq ($p < 0.05$) and passed the Intensity Difference filter in SeqMonk.

ACCESSION NUMBERS

The accession number for the ChIP-seq and RNA-seq data reported in this paper is GEO: GSE86814.

SUPPLEMENTAL INFORMATION

Supplemental Information includes Supplemental Experimental Procedures, four figures, two tables and can be found with this article online at http://dx.doi.org/10.1016/j.celrep.2016.09.050.

AUTHOR CONTRIBUTIONS

R.T.T. and I.A.T. performed all experiments and, together with P.J.L., analyzed the data and wrote the paper. R.A. prepared and analyzed mass spectrometry samples, and G.D. contributed essential reagents.

ACKNOWLEDGMENTS

We are grateful to CIMR core facilities: K. Jayawardena and Y. Umrania for mass spectrometry and R. Schulte and his team for fluorescence-activated cell sorting (FACS). This work was supported by the Wellcome Trust through a Principal Research Fellowship to P.J.L. (101835/Z/13/Z) and a Ph.D. studentship to I.A.T. The CIMR is in receipt of a Wellcome Trust strategic award.

Received: June 1, 2016
Revised: August 19, 2016
Accepted: September 15, 2016
Published: October 11, 2016

REFERENCES

Basavapathruni, A., Gureasko, J., Porter Scott, M., Hermans, W., Godbole, A., Leland, P.A., Boriack-Sjodin, P.A., Wigle, T.J., Copeland, R.A., and Riera, T.V. (2016). Characterization of the enzymatic activity of SETDB1 and its 1:1 complex with ATF7IP. Biochemistry *55*, 1645–1651.

Becker, J.S., Nicetto, D., and Zaret, K.S. (2016). H3K9me3-dependent heterochromatin: barrier to cell fate changes. Trends Genet. *32*, 29–41.

Ceol, C.J., Houvras, Y., Jane-Valbuena, J., Bilodeau, S., Orlando, D.A., Battisti, V., Fritsch, L., Lin, W.M., Hollmann, T.J., Ferré, F., et al. (2011). The histone methyltransferase SETDB1 is recurrently amplified in melanoma and accelerates its onset. Nature *471*, 513–517.

Fei, Q., Shang, K., Zhang, J., Chuai, S., Kong, D., Zhou, T., Fu, S., Liang, Y., Li, C., Chen, Z., et al. (2015). Histone methyltransferase SETDB1 regulates liver cancer cell growth through methylation of p53. Nat. Commun. *6*, 8651.

Fujita, N., Watanabe, S., Ichimura, T., Ohkuma, Y., Chiba, T., Saya, H., and Nakao, M. (2003). MCAF mediates MBD1-dependent transcriptional repression. Mol. Cell. Biol. *23*, 2834–2843.

Huang, W., Sherman, B.T., and Lempicki, R.A. (2009). Systematic and integrative analysis of large gene lists using DAVID bioinformatics resources. Nat. Protoc. *4*, 44–57.

Ichimura, T., Watanabe, S., Sakamoto, Y., Aoto, T., Fujita, N., and Nakao, M. (2005). Transcriptional repression and heterochromatin formation by MBD1 and MCAF/AM family proteins. J. Biol. Chem. *280*, 13928–13935.

Koch, C.M., Honemann-Capito, M., Egger-Adam, D., and Wodarz, A. (2009). Windei, the *Drosophila* homolog of mAM/MCAF1, is an essential cofactor of the H3K9 methyl transferase dSETDB1/Eggless in germ line development. PLoS Genet. *5*, e1000644.

Kouzarides, T. (2007). Chromatin modifications and their function. Cell *128*, 693–705.

Lerdrup, M., Johansen, J.V., Agrawal-Singh, S., and Hansen, K. (2016). An interactive environment for agile analysis and visualization of ChIP-sequencing data. Nat. Struct. Mol. Biol. *23*, 349–357.

Liu, L., Ishihara, K., Ichimura, T., Fujita, N., Hino, S., Tomita, S., Watanabe, S., Saitoh, N., Ito, T., and Nakao, M. (2009). MCAF1/AM is involved in Sp1-mediated maintenance of cancer-associated telomerase activity. J. Biol. Chem. *284*, 5165–5174.

Matsui, T., Leung, D., Miyashita, H., Maksakova, I.A., Miyachi, H., Kimura, H., Tachibana, M., Lorincz, M.C., and Shinkai, Y. (2010). Proviral silencing in embryonic stem cells requires the histone methyltransferase ESET. Nature *464*, 927–931.

Minkovsky, A., Sahakyan, A., Rankin-Gee, E., Bonora, G., Patel, S., and Plath, K. (2014). The Mbd1-Atf7ip-Setdb1 pathway contributes to the maintenance of X chromosome inactivation. Epigenetics Chromatin *7*, 12.

Mozzetta, C., Boyarchuk, E., Pontis, J., and Ait-Si-Ali, S. (2015). Sound of silence: the properties and functions of repressive Lys methyltransferases. Nat. Rev. Mol. Cell Biol. *16*, 499–513.

Rodriguez-Paredes, M., Martinez de Paz, A., Simó-Riudalbas, L., Sayols, S., Moutinho, C., Moran, S., Villanueva, A., Vázquez-Cedeira, M., Lazo, P.A., Carneiro, F., et al. (2014). Gene amplification of the histone methyltransferase SETDB1 contributes to human lung tumorigenesis. Oncogene *33*, 2807–2813.

Rowe, H.M., Jakobsson, J., Mesnard, D., Rougemont, J., Reynard, S., Aktas, T., Maillard, P.V., Layard-Liesching, H., Verp, S., Marquis, J., et al. (2010). KAP1 controls endogenous retroviruses in embryonic stem cells. Nature *463*, 237–240.

Sun, Q.-Y., Ding, L.-W., Xiao, J.-F., Chien, W., Lim, S.-L., Hattori, N., Goodglick, L., Chia, D., Mah, V., Alavi, M., et al. (2015). SETDB1 accelerates tumourigenesis by regulating the WNT signalling pathway. J. Pathol. *235*, 559–570.

Sun, Y., Wei, M., Ren, S.-C., Chen, R., Xu, W.-D., Wang, F.-B., Lu, J., Shen, J., Yu, Y.-W., Hou, J.-G., et al. (2014). Histone methyltransferase SETDB1 is required for prostate cancer cell proliferation, migration and invasion. Asian J. Androl. *16*, 319–324.

Tchasovnikarova, I.A., Timms, R.T., Matheson, N.J., Wals, K., Antrobus, R., Göttgens, B., Dougan, G., Dawson, M.A., and Lehner, P.J. (2015). GENE SILENCING. Epigenetic silencing by the HUSH complex mediates position-effect variegation in human cells. Science *348*, 1481–1485.

Wang, H., An, W., Cao, R., Xia, L., Erdjument-Bromage, H., Chatton, B., Tempst, P., Roeder, R.G., and Zhang, Y. (2003). mAM facilitates conversion by ESET of dimethyl to trimethyl lysine 9 of histone H3 to cause transcriptional repression. Mol. Cell *12*, 475–487.

Waterfield, M., Khan, I.S., Cortez, J.T., Fan, U., Metzger, T., Greer, A., Fasano, K., Martinez-Llordella, M., Pollack, J.L., Erle, D.J., et al. (2014). The transcriptional regulator Aire coopts the repressive ATF7ip-MBD1 complex for the induction of immunotolerance. Nat. Immunol. *15*, 258–265.

Wong, C.-M., Wei, L., Law, C.-T., Ho, D.W.-H., Tsang, F.H.-C., Au, S.L.-K., Sze, K.M.-F., Lee, J.M.-F., Wong, C.C.-L., and Ng, I.O.-L. (2016). Up-regulation of histone methyltransferase SETDB1 by multiple mechanisms in hepatocellular carcinoma promotes cancer metastasis. Hepatology *63*, 474–487.

Yang, B.X., El Farran, C.A., Guo, H.C., Yu, T., Fang, H.T., Wang, H.F., Schlesinger, S., Seah, Y.F., Goh, G.Y., Neo, S.P., et al. (2015). Systematic identification of factors for provirus silencing in embryonic stem cells. Cell *163*, 230–245.

Cell Reports, Volume *17*

Supplemental Information

ATF7IP-Mediated Stabilization of the Histone Methyltransferase SETDB1 Is Essential for Heterochromatin Formation by the HUSH Complex

Richard T. Timms, Iva A. Tchasovnikarova, Robin Antrobus, Gordon Dougan, and Paul J. Lehner

SUPPLEMENTAL FIGURES

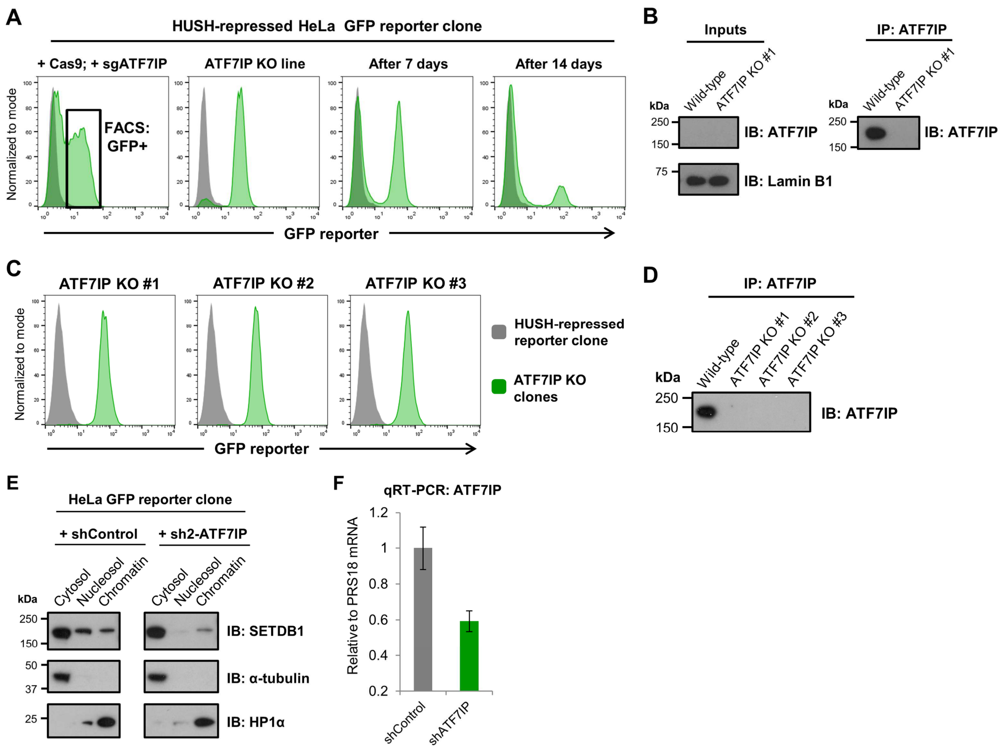

Figure S1. Generation of ATF7IP knockout HeLa clones through CRISPR/Cas9-mediated gene disruption, Related to Figures 1 and 2.

(**A**) A HeLa clone harboring a HUSH-repressed GFP reporter was transfected with plasmids encoding Cas9 and a pool of three sgRNAs targeting ATF7IP. The resulting GFP$^+$ cells exhibiting functional loss of ATF7IP were isolated by FACS, yielding a polyclonal population of ATF7IP knockout cells of ~95% purity. The ATF7IP knockout cells in the population were rapidly overgrown by the remaining wild-type cells, however, necessitating the derivation of individual single cell ATF7IP knockout clones.

(**B-D**) Validation of the ATF7IP knockout HeLa clones. Our antibody against ATF7IP (Bethyl Laboratories, A300-169A) was unable to detect ATF7IP protein in wild-type cells in direct immunoblot analysis; however, we were able to detect ATF7IP by immunoblot following a prior immunoprecipitation step (B). We used this immunoprecipitation-immunoblot assay to verify a loss of ATF7IP protein expression in three independent GFP$^+$ ATF7IP knockout clones (C,D).

(**E-F**) Destabilization of nuclear SETDB1 following shRNA-mediated depletion of ATF7IP. HeLa cells were transduced with an expression vector encoding either a control shRNA or an shRNA against ATF7IP, and SETDB1 levels were assessed by immunoblot following subcellular fractionation (E). Knockdown efficiency was assessed by quantitative reverse transcription PCR (qRT-PCR) (F); error bars represent the standard deviation of three qPCR technical replicates.

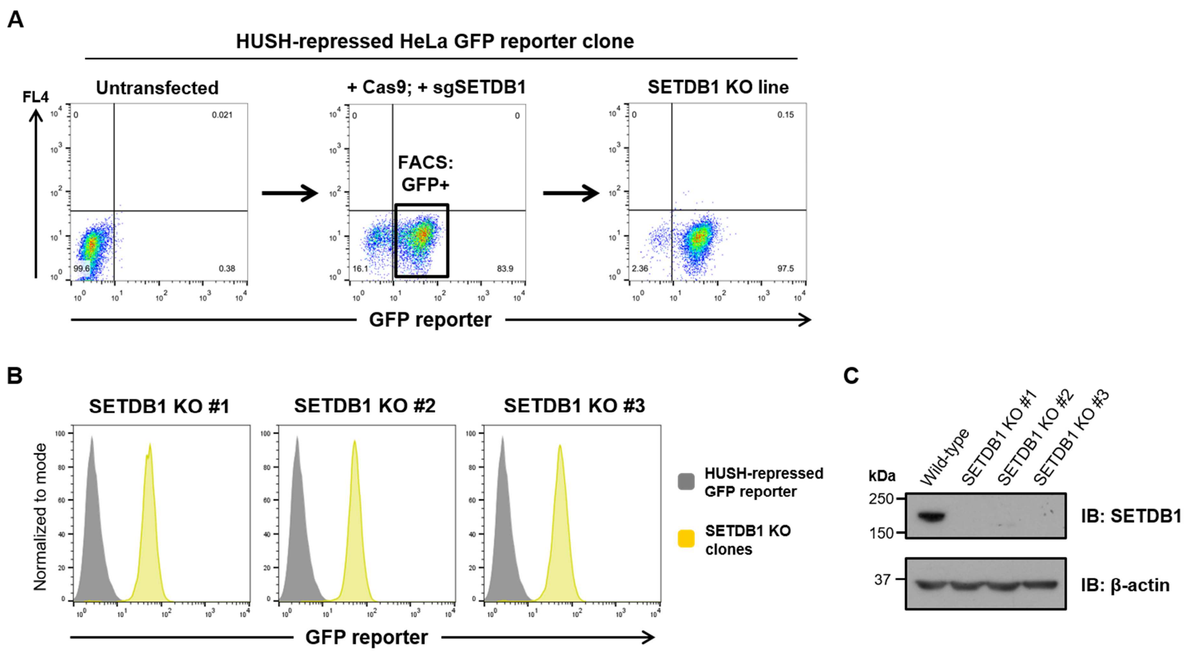

Figure S2. Generation and validation of SETDB1 knockout clones, Related to Figure 2.

(**A,B**) Generation of SETDB1 knockout clones through CRISPR/Cas9-mediated gene disruption. A HeLa clone harboring a HUSH-repressed GFP reporter was transfected with plasmids encoding Cas9 and a pool of four sgRNAs targeting SETDB1. The resulting GFP^+ cells exhibiting functional loss of SETDB1 were isolated by FACS, yielding a population of SETDB1 knockout cells (A) from which three independent GFP+ single cell clones were derived (B).

(**C**) Immunoblot validation of a lack of SETDB1 protein expression in the three knockout clones.

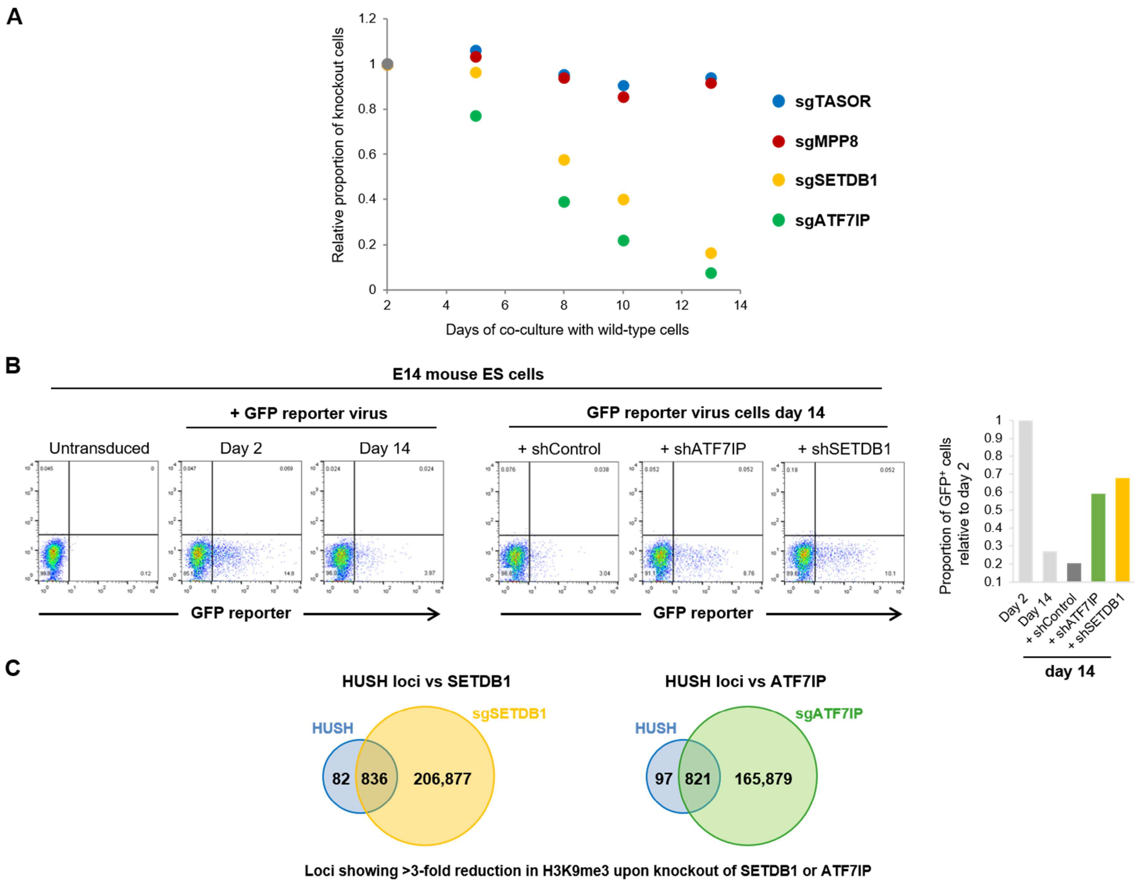

Figure S3. Loss of ATF7IP has similar phenotypic consequences to loss of SETDB1, Related to Figures 3 and 4.

(**A**) Ablation of ATF7IP impairs the growth of HeLa cells to a similar extent to ablation of SETBD1. Wild-type HeLa cells were mixed together with an approximately equal proportion of either ATF7IP or SETDB1 knockout reporter cells. As the knockout cells are GFP^+ owing to derepression of the GFP reporter, dilution of the knockout cells from the population could be followed by flow cytometry. In contrast, cells lacking the HUSH subunits TASOR and MPP8 did not display a significant growth defect compared to the parental wild-type cells.

(**B**) Knockdown of ATF7IP impairs the repression of exogenous viral constructs in murine ES cells to a similar extent to knockdown of SETDB1. mESCs were transduced with a lentiviral vector encoding GFP, which was progressively silenced over the course of a few days. Reactivation of the GFP reporter did not occur upon transduction with a control shRNA, but an increase in the proportion of GFP+ cells was observed upon transduction with shRNAs targeting either SETDB1 or ATF7IP.

(**C**) SETDB1 and ATF7IP are responsible for H3K9me3 deposition at the vast majority of genomic loci at which the HUSH complex acts. Previously we identified 918 genomic loci at which knockout of either TASOR, MPP8 or Periphilin resulted in a significant decrease in H3K9me3 levels (Tchasovnikarova et al., 2015): 836 of these loci also exhibit a >3-fold reduction in H3K9me3 upon knockout of SETDB1, as do 821 of these loci upon knockout of ATF7IP.

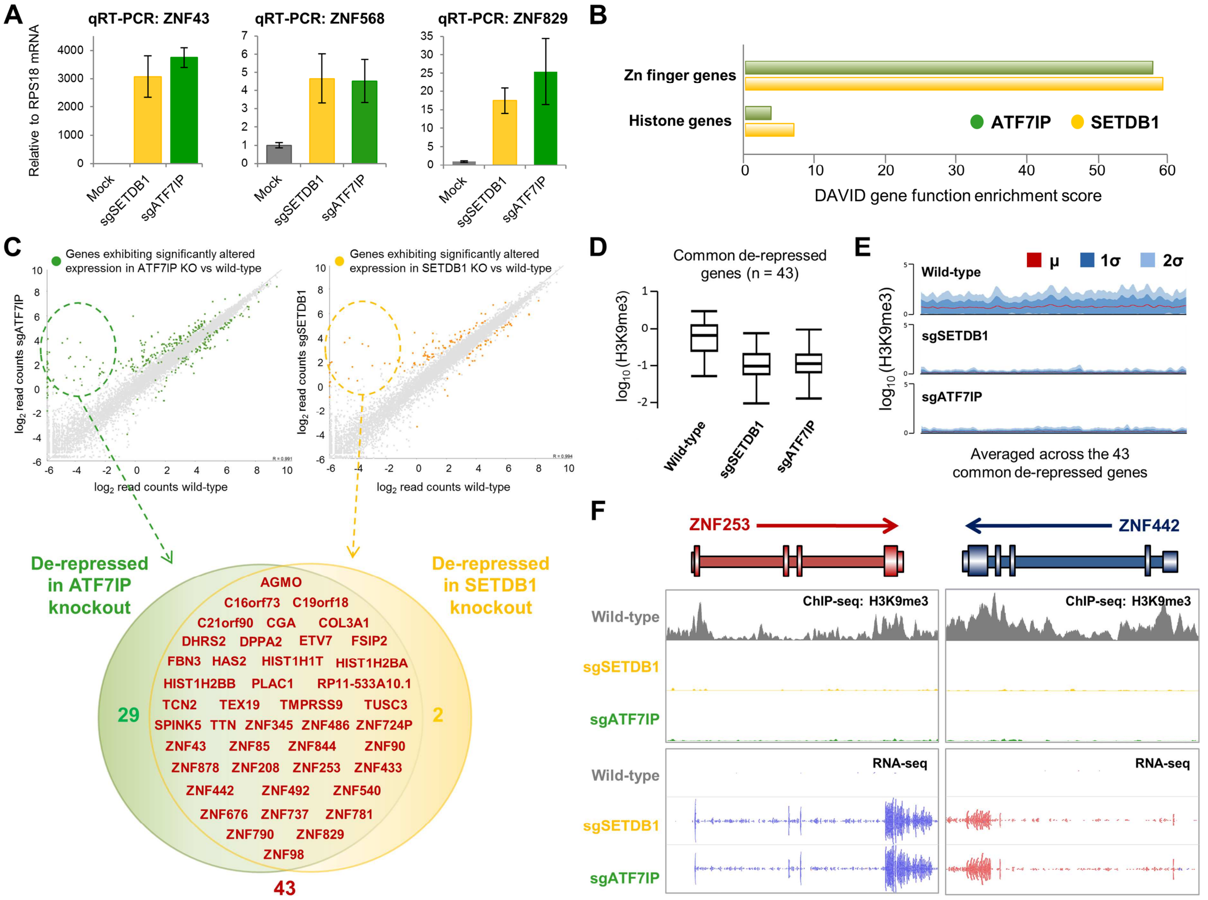

Figure S4. Loss of ATF7IP and SETDB1 has a similar impact on the transcriptome, Related to Figure 4.

(**A**) Validation of the RNA-seq data by qRT-PCR at three example KRAB-ZNF genes. Error bars represent the standard deviation of three qPCR technical replicates.

(**B**) Functional classification of the genes upregulated in ATF7IP and SETDB1 knockout cells using DAVID.

(**C**) Identification of genes that were lowly or not expressed in wild-type cells but which were significantly upregulated upon knockout of ATF7IP or SETDB1. This generated a common set of 43 derepressed genes.

(**D-F**) Transcriptional derepression upon loss of ATF7IP or SETDB1 results from a loss of H3K9me3 deposition. H3K9me3 levels across the common set of 43 upregulated genes were significantly reduced upon ATF7IP or SETDB1 knockout (D,E). H3K9me3 ChIP-seq and RNA-seq traces for two example zinc finger genes are shown in (F).

SUPPLEMENTAL TABLES

Table S1. Putative SETDB1-interacting proteins identified by mass spectrometry, Related to Figure 1.

Uniprot Accession	Protein	Number of peptides: Wild-type	Number of peptides: SETDB1 KO	Mw (kDa)
G3V1U0	Activating transcription factor 7 interacting protein (ATF7IP)	11	0	136.23
O00160	Unconventional myosin-If (MYO1F)	10	0	124.77
Q96C19	EF-hand domain-containing protein D2 (EFHD2)	6	0	26.68
P07195	L-lactate dehydrogenase B chain (LDHB)	4	0	36.61
Q86T24	Transcriptional regulator Kaiso (ZBTB33)	3	0	74.43

Table S2. Oligonucleotide sequences, Related to all Figures.

**sgRNAs**	**20 bp targeting sequence (5'-3')**
sg1-ATF7IP	GAGGCAGCATCACTAGAGGA
sg2-ATF7IP	ACTAGAGGCTATATCATCAG
sg3-ATF7IP	GGATGGCTCTGTAGAAGCTG
sg1-SETDB1	TGGATGCATCATCAAAGAGT
sg2-SETDB1	AACTCTTTGATGATGCATCC
sg3-SETDB1	GCTCTGAGGACGAATCTTCC
sg4-SETDB1	CAGAACTCCAAAAGACCAGA
**shRNAs**	**Forward oligonucleotide sequence (5'-3')**
shControl	GATCCGGGTATCGACGATTACAAATTCAAGAGATTTGTAATCGTCGATACCCTTTTTTG
sh1-ATF7IP	GATCCGGAAGAGGTGAATGGCATTTTCAAGAGAAATGCCATTCACCTCTTCCTTTTTTG
sh2-ATF7IP	GATCCGCCAAGATAGCCAGGTTAACTTCAAGAGAGTTAACCTGGCTATCTTGGTTTTTTG
shATF7IP (mouse)	GATCCGCGACGAACACTTAGACCAATTCAAGAGATTGGTCTAAGTGTTCGTCGTTTTTTG
shSETDB1 (mouse)	GATCCGAGGAACTTCGTCAGTACATTCAAGAGATGTACTGACGAAGTTCCTCTTTTTTG
**qPCR primers**	**Forward oligonucleotide sequence (5'-3')**
ATF7IP_F	ATGTGGTTGTTTGAGGCACT
ATF7IP_R	CCTTGCGAGGAACTGTTATG
RPS18_F	GCGGCGGAAAATAGCTTG
RPS18_R	TGAGTTCTCCCGCCCTCTTG
ZNF37A_F	CATTCTGAAGAGGAACCTTCTG
ZNF37A_R	TTTCTGATGCCAAATGAGGT
ZNF221_F	AAGGCATGATTTCACCTTCA
ZNF221_R	CTGCCTCTTTGAATGTGGTC
ZNF594_F	GCCTGGAGATTCTGAAGACA
ZNF594_R	CACATTCCTGGGTGATTTGT
ZNF43_F	GACCCTCATACCTGGAACCT
ZNF43_R	GTGCAGGCTGAAGAAGAGTC
ZNF568_F	CAGGCCTCTTGAAACAGTGA
ZNF568_R	TTTGAGCAGGTTTCATTTGC
ZNF829_F	GAGGATACCGAAGACCGATG
ZNF829_R	AGAGAGGTTTCCATGTTGGC

SUPPLEMENTAL EXPERIMENTAL PROCEDURES

Cell culture. HeLa cells were obtained from the ECACC and were grown in RPMI 1640 plus 10% FCS and penicillin/streptomycin, and, where stated, treated with the proteasome inhibitor Bortezomib (NEB) at a final concentration of 20 nM for 40 h. HEK 293ET cells, a generous gift from Dr. Felix Randow (MRC-LMB, Cambridge, UK), were cultured in IMDM plus 10% fetal calf serum (FCS) and penicillin/streptomycin. E14 mouse embryonic stem cells were a generous gift from Prof. Berthold Göttgens (CIMR, Cambridge, UK) and were cultured on gelatinized plates in KnockOut DMEM (Thermo Fisher Scientific) supplemented with 15% fetal bovine serum, 1000 U/ml LIF (Millipore), 2 mM L-glutamine, 0.1 mM β-mercaptoethanol and penicillin/streptomycin.

Antibodies. Primary antibodies used were as follows: rabbit α-SETDB1 (Proteintech, 11231-1-AP, 1:5000), rabbit α-ATF7IP (Bethyl Laboratories, A300-169A, 1:1000), rabbit α-GAPDH (Cell Signaling, #2118, 1:5000), mouse α-α-tubulin (Cell Signaling, #3873, 1:5000), rabbit α-HP1α (Cell Signaling, #2616, 1:5000), mouse α-β-actin (Sigma-Aldrich, A5316; 1:10000) and goat α-lamin B1 (SantaCruz Biotech, sc-20682, 1:1000).

CRISPR/Cas9-mediated gene disruption. Oligonucleotides (obtained from Sigma-Aldrich) encoding the for top and bottom strands of the sgRNA were phosphorylated using T4 polynucleotide kinase (NEB), annealed by heating to 95°C followed by slow cooling to room temperature, and then cloned into either the dual Cas9/sgRNA expression vector pSpCas9(BB)-2A-Puro (Addgene #48139, deposited by Dr. Feng Zhang (Ran et al., 2013)) or the lentiviral sgRNA expression vector pKLV-U6gRNA(BbsI)-PGKpuro2ABFP (Addgene

#50946, deposited by Dr. Kosuke Yusa (Koike-Yusa et al., 2014)). The sgRNAs expressed from pSpCas9(BB)-2A-Puro were transfected into HeLa cells using TransIT-HeLaMONSTER (Mirus) according to the manufacturer's protocol, and the transfected cells enriched by two days of puromycin selection starting 24 hours after transfection. For lentiviral CRISPR/Cas9-mediated gene disruption, cells were first transduced with the Cas9 expression vector pHRSIN-P_{SFFV}-Cas9-P_{PGK}-Hygro, selected on hygromycin, and then subsequently transduced with the lentiviral sgRNA expression vector and selected using puromycin. All sgRNA sequences are detailed in Table S2.

RNA interference. Lentiviral expression of shRNA constructs was achieved using the pHR-SIREN vector, with hairpins cloned in as BamHI-EcoRI fragments. All shRNA sequences are detailed in Table S2.

Lentiviral expression. Lentivirus was produced through the triple transfection of HEK 293ET cells with three plasmids: the lentiviral transfer vector, and the packaging plasmids pCMVΔR8.91 and pMD.G. Transfection was achieved using the TransIT-293 reagent (Mirus) as recommended by the manufacturer. The viral supernatant was collected 48 h later, passed through a 0.45 μm filter, and target cells transduced by spin infection at 700 x *g* for 60 min. The viral vector used to transduce mouse ES cells was a pHRSIN-based lentivirus encoding GFP from the Rous sarcoma virus (RSV) LTR promoter (pHRSIN-P_{RSV}-GFP).

Flow cytometry. Harvested cells were washed once with PBS and then fixed in 1% formaldehyde prior to analysis on a FACSCalibur instrument (BD). Cell sorting was carried out on an Influx cell sorter (BD).

Immunoblotting. Cells were lysed in 1% SDS in TBS plus protease inhibitor cocktail (Roche) at room temperature for 30 min. Samples were then heated in SDS sample buffer for 10 min at 70°C, separated by SDS-PAGE, and transferred to a PVDF membrane (Millipore). The membranes were blocked by incubation in 5% milk in PBS + 0.2% Tween-20 for at least 30 min, and then probed overnight at 4°C with the indicated primary antibodies. Following 3 x 5 min washes with PBS + 0.2% Tween-20, membranes were incubated with HRP-conjugated secondary antibodies for 45 min at room temperature and reactive bands visualized using ECL western blotting substrate, West Pico or West Dura (Thermo Fisher Scientific).

Quantitative reverse transcription PCR (qRT-PCR). RNA was extracted from HeLa cells with the RNeasy Plus kit (Qiagen) and then converted into cDNA using Super RT reverse transcriptase (HT Biotechnology) with a poly(d)T primer following the manufacturer's instructions. Samples were analyzed on an ABI 7500 Real Time PCR System (Applied Biosystems) using SYBR green PCR mastermix (Life Technologies). PCR reactions were performed in a total reaction volume of 25 μl, using 20 ng of cDNA, 10 μl of SYBR green PCR mastermix and 0.2 μM of forward and reverse primers. Cycling parameters were 50°C for 2 min and 95°C for 5 min, with a subsequent 40 cycles of 95°C for 15 s, 58°C for 1 min. Primer sequences are detailed in Table S2.

Statistical methods. All qPCR data is represented as the mean +/- standard deviation of three technical replicates. For the ChIP-qPCR data presented in Figure 3D, individual dots represent 1 kb genomic intervals; only intervals with greater than 40 reads in one of the samples were used for calculating the Pearson correlation coefficient. For the RNA-seq data presented in Figure 4B-E, the highlighted genes exhibited differential expression as determined by DEseq ($P < 0.05$) and also passed the Intensity Difference filter in SeqMonk.

SUPPLEMENTAL REFERENCES

Koike-Yusa, H., Li, Y., Tan, E.-P., Velasco-Herrera, M.D.C. and Yusa, K. (2014). Genome-wide recessive genetic screening in mammalian cells with a lentiviral CRISPR-guide RNA library. Nat. Biotechnol. *32*, 267–73.

Ran, F.A., Hsu, P.D., Wright, J., Agarwala, V., Scott, D.A. and Zhang, F. (2013). Genome engineering using the CRISPR-Cas9 system. Nat. Protoc. *8*, 2281–308.
